# Canadian adolescent perceptions and knowledge about the social determinants of health: an observational study of Kingston, Ontario youth

**DOI:** 10.1186/1471-2458-13-781

**Published:** 2013-08-27

**Authors:** Kelly E Kenney, Spencer Moore

**Affiliations:** 1 School of Kinesiology and Health Studies, Queen’s University, 28 Division Street, Kingston, ON K7L 4M7, Canada; 2 Department of Public Health Sciences, Queen’s University, 28 Division Street, Kingston, ON K7L 4M7, Canada

**Keywords:** Social determinants of health, Adolescents, Education, Knowledge translation

## Abstract

**Background:**

Upstream social determinants of health (SDH) have become widely acknowledged as lying at the root of poor health outcomes in Canada and globally. The Commission on the Social Determinants of Health maintains that educating the public about the SDH is a key step towards population health equity. Little is known about adolescent perceptions of the determinants of health. Curriculum in Ontario is lacking in SDH content, placing a much greater emphasis on individual, lifestyle behaviors, such as diet, physical activity, and safe sex practices. Identifying a gap in SDH knowledge within the adolescent population is required to advocate for health curriculum revision to include SDH material.

**Methods:**

Student sociodemographic information was obtained through a self-administered questionnaire. Concept mapping exercises were used to determine students’ knowledge of the determinants of health and the SDH. Knowledge was approximated by the relative number of SDH concepts present in student maps. Poisson regression analysis was used to determine correlations between sociodemographic characteristics and SDH knowledge.

**Results:**

Concept maps indicated that students attributed their health primarily to physical determinants versus social determinants; 44% of maps contained no SDH content. Statistical analyses indicated that students’ SDH knowledge varied by their relative socioeconomic status (SES).

**Conclusions:**

Findings suggest that 1) there is an SDH knowledge gap in the adolescent population, and 2) an inequity in adolescent SDH knowledge exists across socio-economic factors. Current Ontario health curriculum requires revision to include SDH material, which will require greater communication and collaboration from both educational institutions and health agencies in Canada.

## Background

The social determinants of health (SDH) consist of the structural drivers and daily life conditions that influence a person’s health status [1-4]. The SDH include such factors as income and educational status, employment opportunities, housing conditions, social exclusion, racism, and inequality. Evidence of the impact of such factors on health, including mortality and morbidity, occurs at multiple levels of influence and through a multitude of pathways [3,4]. Despite a rich history of health promotion in Canada, there has been very little research examining the Canadian public’s perceptions of the SDH [5]. When conducted, studies usually focus on adults rather than adolescents. Research investigating adolescent health knowledge has tended to focus on adolescent knowledge about risky health behaviours (e.g. tobacco and alcohol use, sedentary activity, poor nutrition, safe sexual practices) and not about their understandings of the SDH. Less is known about the behavioural factors that adolescents perceive significant to their health, and whether they even recognize the role of the SDH on health. In short, as expressed by Woodgate and Leach (2010), the focus has been on what youth do, not what they think and feel [6]. Health-related values, attitudes and behaviours formed in adolescence have been shown to predict significant health risks in adulthood, including issues of social and economic disadvantage [6,7]. Knowing what youth perceive as critical health determinants can help practitioners to identify gaps in health education curriculum. This subsequently can contribute to the design of programs that will foster a broader understanding of health, emphasizing the impact of the social and economic environment alongside lifestyle behaviours on health. This is an integral message within the Ottawa Charter for Health Promotion – a document that has guided health promotion practices globally since its publication [8,9].

Acting effectively to address the SDH requires in part a greater public understanding and awareness of the SDH [1]. When asked to identify the most important factors that contribute to good health, Canadian adults tend to attribute greater influence to personal health behaviours, such as physical activity and diet than to social and economic conditions [5]. According to the Canadian Public Health Institute (CPHI), only one in three Canadian adults believed that social, economic, and environmental conditions had an impact on health [5]. The research that has explored Canadian adolescents’ perceptions of health has tended to show similar results as those found in Canadian adults. Youth had broad understandings of health, attributing health to a variety of distinct domains including physical, mental, social and environmental health. However, personal behaviors and practices, specifically exercise and diet, were seen as the main determinants of health [6]. Youth descriptions of the connections between the SDH and health outcomes have been described as vague and disjointed, suggesting a noticeable lack of understanding [6]. The lack of relevant literature on how Canadian adolescents perceive the determinants of health has created a noticeable gap in health education and promotion research.

Enhancing education and public awareness of the SDH is a step towards action in reducing health inequalities [10]. The Commission in the Social Determinants of Health (CSDH) (2008) recommends that greater knowledge of the SDH should be encouraged outside the medical and health research community. The exposure to health information may be most beneficial in youth, as adolescents have high learning capabilities, as well as great potential to use health information to their advantage now *and* as they age [11,12]. Positive health values, attitudes, and behaviours that are formed at young ages are likely to continue to be encouraged and built upon as youth transition into adulthood [7]. Youth report that their exposure to health information is heavily weighted towards lifestyle behaviours [6]. The majority of health information is delivered to adolescents through the health education curriculum within schools; educational policies are thus in a powerful position to influence adolescent knowledge and understanding of health and the SDH [13]. Review of Ontario guidelines for secondary school education reveals that health curriculum weighs heavily towards lifestyle behaviours [14]. Current health education is relatively void of SDH theory and content, and reinforces the biomedical model of health within secondary school health programs.

Our research on adolescent perceptions of health is a step towards determining a potential knowledge gap in Ontario youth concerning the SDH. The main purpose of this study was thus to evaluate what determinants of health adolescents tend to associate with health, as well as to assess their relative understanding of the influence of the SDH on health in comparison to physical determinants. The research questions which guided our study and analysis were 1) What determinants (social, behavioural, and physical) do Ontario high school students associate with health?, 2) To what degree do Ontario high school students know about the SDH?, and 3) Are there any socioeconomic differences in students’ knowledge of the SDH? Conclusions drawn will inform suggestions for future Ontario health curriculum modification to better educate students about the SDH alongside mainstream physical determinants.

## Methods

### Sample and participants

Kingston is located in southeastern Ontario, Canada. In 2011, the city was home to approximately 123,363 people, with a median age of just over 40 years old [15]. Private households comprised 27% of the population, 17% of which were single-parent families [15]. According to 2007 Canada Census data, nearly 25% of Kingston males and 23.5% of females were university educated, which is above the provincial average for both sexes [16]. Kingston was selected as the site for this study due to its proximity to the researchers’ institutional locations. The Limestone District School Board (LDSB) is one of two school boards operating within Kingston. The LDSB comprises eleven secondary schools located in Kingston and the surrounding areas. Two of the schools are remote (approximately 75 km and 111 km away from the LDSB main offices) and were excluded from the study due to travel constraints. Initial contact was made with eligible schools in December 2010; by February 2011, three schools showed interest in participating: (1) Kingston Collegiate Vocational College (KCVI), (2) La Salle Secondary School (LSS), and (3) Napanee District Secondary School (NDSS). The study targeted the health and physical education classes within each school. In total, six health and physical education classes took part in the study. At the time of the project, a total of 133 students were registered in the six health and physical education classes. SDH educational sessions took place with students in these classes. Students could take part in the educational session without consent but were required to have a signed consent from their parents to be research participants, i.e., complete the questionnaire and submit their concept map for analysis. Approval to conduct research in the Kingston secondary school system was granted from the LDSB and the Queen’s University General Research Ethics Board (GREB) in August of 2010.

### Measures

#### Instruments

The students completed a self-administered questionnaire consisting of six items used to assess participants’ socio-demographic background. The items included the students’ sex, grade, primary and secondary schools of attendance, length of residency in the Kingston area, number of siblings, and mother’s educational attainment. Following completion of the questionnaire, students were instructed on how to create a simple concept map to demonstrate their individual understandings of the determinants of health. Concept mapping is one way in which student levels of knowledge and understanding can be extracted, documented, and evaluated. Concept mapping is a creative and interactive method for visually organizing thoughts, and is commonly used in teaching, learning and assessment in educational settings [17-20]. We viewed the use of concept mapping in this study as helping encourage meaningful and critical thinking in students and enabling them to identify and appraise what they know about SDH. Students were encouraged to think about how the health concepts that they identified were related to one another, and to illustrate connections where relationships were thought to exist. Each map began with the word “health” as the focal point; students were given 10–15 minutes to build their maps. Knowledge of health and of the SDH was evaluated by the enumeration of different types of concepts present in student maps [21,22].

### Socio-demographic variables

Socio-demographic and -economic variables included participants’ gender, the number of siblings, the name of the elementary school that they attended, grade level, length of residency in Kingston, and their mother’s level of educational attainment. The investigator recorded the name of the participant’s high school. Socioeconomic status (SES) was based on students’ reports of maternal educational attainment as being (1) less than high school, (2) high school or equivalent, (3) college diploma (4) university degree, or (5) advanced university degree (MA, MSc, PhD, MD). (In Canada, a college diploma represents a two-year practice based education whereas a university degree refers to a three or four-year Bachelors degree program). Responses were later dichotomized into either having attained a university education or not. Maternal education was chosen to estimate SES because it has been shown to be a stronger predictor of childhood health than paternal education [23,24].

### Map measures

To evaluate the SDH knowledge of students and the determinants they most frequently associated with health, three distinct measures from the concept maps were developed: (1) diversity, (2) frequency, and (3) knowledge about the SDH. To assess diversity, the concepts found in each map were sorted into 12 different conceptual categories (Appendix 1). Diversity reflects the number of different conceptual categories that a student included in their map, and was categorized into low (<4 categories), medium (4–7 categories), and high (8+) levels. Frequency reflects the number of concepts within each category drawn on a map. Knowledge about the SDH was determined by counting SDH-related content within each map.

A primary coder classified the different concepts found within each of the 62 maps into the 12 categories and determined whether any of the concepts were related to the SDH. Face validity and content validity was determined for each concept category by defining each using definitions established by sources such as the Merriam-Webster Dictionary, the World Health Organization (WHO), the Canadian Society of Exercise Physiology, and relevant peer-reviewed sources [4,25,26]. To assess the reliability of the classification method, a second coder classified 20% of the concepts found within the maps into separate health categories based on criteria developed by the primary coder. The Cohen’s kappa inter-coder reliability coefficient was 0.89, which represents substantial to near perfect agreement. Discrepancy occurred primarily over the concept of “fit” and to whether “fit” related moreso to physical activity or personal appearance (2.7%), and to whether concepts such as “determination” and “self control” should be considered mental health or personality traits (2.7%).

### Analysis

Given that our outcome variable was a count of the number of SDH concepts, bivariate and multivariate Poisson regression analyses were conducted to estimate the association between socio-demographic and –economic variables and SDH map content. Bivariate regression was used to determine which variables to include in the finale multivariable regression models. Due to limited sample size, only variables found to be significantly related to SDH map content in a bivariate regression were included in the final multivariate regression. Analyses were performed using Stata version 12.

## Results

### Survey content

#### Sample characteristics

Consent return rates were 46.6% for a final sample size of 62 students from three secondary schools in the LSBD in Kingston, Ontario. There was a greater percentage of female (53.2%) than male (46.8%) participants. The majority of the students were in grade 11 (75.8%), followed by grade 12/13 (16.1%) and grade 10 (8.1%). Over 96% of the sample had resided in Kingston since the beginning of their secondary schooling. In terms of maternal education, nearly 53% of the participants’ mothers had a university degree (Table 1).

**Table 1:** Sample socio-demographic and -economic characteristics (n = 62)

***Characteristic***	***No. (%)***
Gender	29 (46.8)
Male	
Female	33 (53.2)
High school	
KCVI	28 (45.2)
LSS	18 (29.0)
NDSS	16 (25.8)
Grade	
Grade 10	5 (8.1)
Grade 11	47 (75.8)
Grade 12	8 (12.9)
Grade 13	2 (3.2)
Mother’s educational attainment	
< University	29 (46.8)
Undergraduate/advanced university degree	33 (53.2)

### Map content

#### Diversity and frequency of the determinants of health

Maps contained an average of 12 concepts (SD = 5.65) with the range from 2–31 concepts. Maps had an average diversity of 5.4 (SD = 1.8) different categories of health. The most comprehensive map contained 10 different categories of health. The most commonly occuring categories of concepts were physical activity, diet/nutrition, and mental health (Table 2).

**Table 2:** Summary statistics for the map concepts in categories 1–12 (n = 62)

***Category***	***Mean frequency***	***Std Dev***	***Minimum***	***Maximum***
(1) Physical activity	2.42	1.53	0	7
(2) Diet/nutrition	2.37	2.04	0	12
(3) Mental	1.92	1.86	0	8
(4) Genetic	0.5	1.05	0	6
(5) Appearance	0.48	1.05	0	6
(6) Substance use	0.23	0.66	0	3
(7) Daily life	0.29	0.55	0	2
(8) Sexual	0.37	0.85	0	4
(9) Medical	0.9	1.34	0	6
(10) Social	1.26	1.93	0	8
(11) Environmental	0.73	1.07	0	5
(12) SDH	2.21	3.0	0	15

### Social determinants of health knowledge

The map with the highest level of SDH knowledge contained 15 SDH concepts. However, students who included many SDH concepts were somewhat repetitive with their use, resulting in larger maps with little variation (e.g., students may have “income”, “money” and “finances” listed as separate concepts, when they represented a common idea). The mean number of SDH concepts on a map was 2.21 (SD = 3.0). The median number of SDH concepts was 0; appoximately 43% of the students had no SDH drawn on their maps.

Bivariate and multivariate poisson regression results indicated that students’ SDH knowledge varied by high school, grade-level, and socioeconomic status (See Table 3). Participant gender, elementary school of attendance, length of residency in Kingston, and number of siblings were not significantly related to SDH content in bivariate analysis, and so were not included in the multivariate regression. The final regression model included the variables: high school of attendance, grade, and maternal education. SDH content was more likely to occur in student concept maps from KCVI than those from LSS (IRR = .53, 95% CI = .31 - .88) or NDSS (IRR = .51, 95% CI = .31 - .87). Students in grade 11 and grade 12/13 were more likely to have SDH concepts in their maps than those in grade 10, respectively (IRR = 4.8, 95% CI = 1.50 – 15.82; IRR = 7.66, 95% CI = 2.38 – 24.60). Students with relatively higher socioeconomic status (estimated by maternal educational attainment of a undergraduate/advanced university degree) were more likely to have SDH concepts in their maps than students with lower socioeconomic status (IRR = 1.61, 95% CI = 1.11 – 2.32).

**Table 3:** Bivariate and multivariate poisson regressions of SDH content and high school of attendance, SDH content and grade level, and SDH content and maternal education (n = 61)

	***Bivariate***		***Multivariable***	
***Variable***	***IRR***	***95% CI***	***IRR.***	***95% CI***
High school of attendance				
KCVI (reference)	---	---	---	---
LSS	.45**	.28 - .71	.53*	..31 - .88
NDSS	.46**	.29 - .72	.51*	..31 - .87
Grade				
10 (reference)	---	---	---	---
11	3.04	.96 – 9.6	4.88**	1.50 – 15.82
12/13	8.0**	2.49 – 25.68	7.66**	2.38 – 24.60
Maternal educational attainment				
< University (reference)	---	---	---	---
Undergrad. /Advanced Univ. Deg.	1.87**	1.3 – 2.67	1.61*	1.11 – 2.32

*p < 0.05, ** p < 0.01.

## Discussion

Health promotion and a focus on the SDH have a rich tradition in Canada. Nevertheless, little is known about adolescent awareness of the SDH or how they are reflected in educational policies targeted at youth. Canadian educational policies appear incongruous with the emphasis that Canadian health promotion puts on the role of the SDH in achieving good health [27]. Health-related values and behaviours developed in adolescence endure through adulthood [7]. Adolescents are and will continue to be key contributors to community health as they grow and take on greater roles in society. Understanding adolescent perceptions of health is necessary to address potential gaps in health curriculum, specifically, in regards to the SDH. The concept map exercise indicated that our sample of adolescents have a fairly broad perception of health, and recognize that a variety of different components contribute to one’s health. Yet, the heavy emphasis placed on the physical determinants of health and lifestyle practices, and the general lack of SDH map content tend to confirm previous findings regarding both adolescent and adult perceptions of health [5,6]. Our study compliments prior qualitative, interview-based research in this area involving Manitoban youth [6] by providing a quantitative perspective and extending the focus to include a sample of Ontario youth of similar age.

The lack of SDH content on the concept maps could reflect the general absence of SDH education within the Ontario secondary school system. The Commission on the Social Determinants of Health (CSDH) maintains that addressing the knowledge gap in the SDH is an essential step in improving population health [10]. There is disconnect between what we know about the SDH and how we act, and addressing that gap should be a priority for educators and policy makers alike [28]. Our analysis indicated that students with lower socioeconomic backgrounds tend to know less about the SDH in comparison to their classmates. Although research in this field is limited, this finding is consistent with other research that has shown among adults that those of lower SES tended to know less about protective health behaviours regarding cancer [29]. Positive correlations between level of general health knowledge and household income and education (common indicators of SES) in adult populations have also been observed [30,31]. This further supports intervention during public school, as it provides students equal opportunity to learn regardless of their socioeconomic background. *Just Health Action (JHA)* has been working since 2004 to develop a health curriculum that educates students throughout the United States about the SDH [32]. Educators have reported success in teaching SDH within the secondary school system, and have supported and advocated for the inclusion of explicit SDH content in health courses [33].

Education on the SDH is essential in order to develop more effective policy and programs to intervene upon them [34]. There is a need to evaluate what youth view as the determinants of health to address potential knowledge gaps through educational and health policies. Recognition that current widespread health issues are the end result of deep-rooted social inequalities has created a shift in public health research, but public educational curriculum guidelines in Ontario have been slow to reflect those recognitions. Substantial, compelling evidence to guide appropriate and effective action on the SDH *already* exists [10]. Expanding mandatory health education to incorporate SDH content is a sound, powerful route to action on the SDH in adolescent populations.

There are a number of limitations to the study. First, data came from a convenience sample of Ontario secondary students taking Health and Physical Education classes and conclusions may not therefore be generalizable to the rest of the school, Kingston, or Ontario as a whole. School demographic data was not available for comparison. Without further research, however, it is difficult to state whether the results over- or underestimate the amount of knowledge that Ontario adolescents have about the SDH. Second, the concept mapping exercise was simplified for study purposes: only a small amount of time was available for concept mapping instruction. Compared to other studies using concept mapping, the maps created by our participants were less evolved, and few contained prepositions, or “linking words” characteristic of other concept maps [35]. Thirdly, we have chosen to interpret the repetition of certain SDH concepts as indicative of low *range* of knowledge of *different* SDH concepts; alternatively, however, the repetition could represent the heightened value that a student places on that concept compared to others. Fourthly, the 46.6% participant consent rate was less than desirable, however the difficulty in obtaining parental consent was anticipated as a barrier before the study began. Lastly, we did not ask students about household income in assessing student SES, and based our variable on maternal education only.

## Conclusion

Despite such limitations, the results of our research are useful in advancing a health educational curriculum within Ontario schools that more accurately reflects and aligns with Canadian health promotion ideals and positions. At the local school board level, administrators need to enforce SDH education within schools, and provide support for educators who are potentially learning and teaching less familiar material. At the provincial level as well, there is a need for greater cross-institutional conversations and planning on the health curriculum within schools. Ensuring that Canadian youth have a comprehensive understanding of health and the upstream social determinants of health is critical in advancing public policy that aims to intervene on the SDH.

## Appendix 1: Health categories

1) PHYSICAL ACTIVITY: Movement that increases heart rate or breathing; any bodily movement produced by skeletal muscles that requires energy expenditure. Physical activity concepts may have included general terms or specific examples of being active or exercising (E.g., exercise, running, strength training, push-ups).

2) DIET/NUTRITION: Diet/nutrition-related concepts may include general terms or specific examples that refer to food, nutrients, and diet (E.g., food, nutrients, carbohydrates, vitamins, eating right).

3) MENTAL HEALTH: a state of well-being in which an individual realizes his or her own abilities, can cope with the normal stresses of life, can work productively and is able to make a contribution to his or her community of or relating to the total emotional and intellectual response of an individual to external reality. Mental health concepts may have included general terms or specific examples of stress, anxiety (E.g., stress, coping, anxiety) or processes which alleviate mental stress (E.g., relaxation, sleep, meditation, etc.). Concepts could have also included self-esteem, self-confidence, self-efficacy, and disposition/mood (e.g., confident, insecure, low/high self-esteem, happy).

4) GENETICS/ PERSONALITY: Genetic/personality concepts may have included general terms or specific examples of characteristics innate to the individual (e.g., genes, personality traits, heredity).

5) APPEARANCE: External show, outward aspect; outward indication or impression. Appearance related concepts may have included general terms or specific examples related to physical appearance (obese, skinny, muscular, fit). May also have referred to body image (good-looking, ugly, pretty, etc.).

6) SUBSTANCE USE/ABUSE: Something (as drugs or alcoholic beverages) deemed harmful and subject to legal restriction. Substance-related concepts may have included general terms or specific examples related to using drugs, alcohol, tobacco, or to any illicit substance (e.g., smoking, drinking, marijuana, addiction).

7) DAILY LIFE: Personal management and skills that were viewed as necessary for everyday activities. May have included general terms or specific examples related to daily living skills, such as hygiene, cleanliness, or mobility issues that directly affect quality of life. May have also included reference to daily routines/schedules (e.g., balance, routine).

8) SEXUAL HEALTH BEHAVIOUR: a state of physical, mental and social well-being in relation to sexuality. Sexual health concepts may have included general terms or specific examples related to sexual health behaviour, safe sex practices, or sexual reproduction (e.g., safe sex, condoms, contraception).

9) BIOMEDICINE/ILLNESS: Of, relating to, or concerned with physicians or the practice of medicine; requiring or devoted to medical treatment. May have included general terms or specific examples related to the medical system (e.g., hospitals, healthcare, medication, doctors, nurses, insurance). Category may have also included reference to illness or disease (e.g., sickness, specific conditions).

10) INTERPERSONAL RELATIONS: The interpersonal interaction between two individuals or localized group of individuals. Concepts within this category included those pertaining to relationships among family, friends, or peers.

11) ENVIRONMENT: The complex of physical, chemical, and biotic factors (as climate, soil, and living things) that act upon an organism or an ecological community and ultimately determine its form and survival. Environmental concepts may have included general terms or specific examples related to physical surroundings (e.g., buildings, recreational space), the natural environment (e.g., air, trees, soil, fresh water), or conditions in the natural environment (e.g., pollution, clean air, pesticide use).

12) SOCIAL DETERMINANTS OF HEALTH: Social circumstances that shape the distribution of money, power and resources at global, national and local levels [4]. SDH concepts may include general terms or specific examples related to income, income inequality, education, unemployment, job security food environment, Aboriginal status, housing, or gender, or race.

## Competing interests

The authors declare that they have no competing interests.

## Authors’ contributions

KK and SM designed the research and contributed to the conceptualization of the study. KK collected the primary data, delivered the educational sessions, conducted data analysis and drafted the initial version of the manuscript. SM gave advice and input on the sessions, data analyses, and edited the manuscript for intellectual content and clarity. Both authors assume responsibility for the final content and approve the manuscript for submission. There are no conflicts of interest to report. Both authors read and approved the final manuscript.

## Pre-publication history

The pre-publication history for this paper can be accessed here:

http://www.biomedcentral.com/1471-2458/13/781/prepub
